# 1,1′-(Butane-1,4-diyl)di-1*H*-imidazole–benzene-1,3,5-triol–water (1/1/1)

**DOI:** 10.1107/S160053680802240X

**Published:** 2008-07-19

**Authors:** Jin-Sheng Gao, Ying-Hui Yu, Guang-Feng Hou

**Affiliations:** a College of Chemistry and Materials Science, Heilongjiang University, Harbin 150080, People’s Republic of China

## Abstract

The asymmetric unit of the title compound, C_10_H_14_N_4_·C_6_H_6_O_3_·H_2_O, contains one mol­ecule of benzene-1,3,5-triol, two half-molecules of 1,1′-butane-1,4-diyldi-1*H*-imidazole (each molecule is centrosymmetric) and one solvent water mol­ecule. In the crystal structure, inter­molecular O—H⋯O and O—H⋯N hydrogen bonds link all mol­ecules into a three-dimensional supra­molecular network.

## Related literature

For background and details of the synthesis of 1,1′-(1,4-butanedi­yl)diimidazole, see: Ma *et al.* (2003 ▶). For the related crystal structure of 1,1′-(1,4-butanedi­yl)diimidazole, see: Yu *et al.* (2008 ▶).
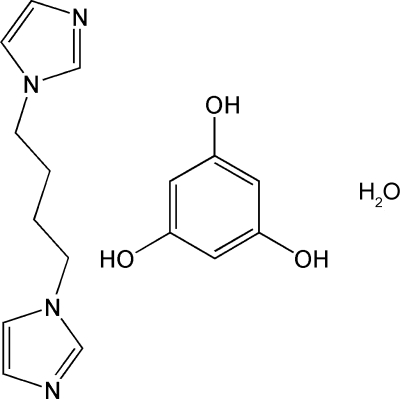


## Experimental

### 

#### Crystal data

C_10_H_14_N_4_·C_6_H_6_O_3_·H_2_O
*M* *_r_* = 334.38
Triclinic, 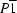

*a* = 7.964 (5) Å
*b* = 8.405 (7) Å
*c* = 14.800 (9) Å
α = 98.40 (3)°
β = 92.93 (2)°
γ = 117.47 (3)°
*V* = 861.5 (10) Å^3^
*Z* = 2
Mo *K*α radiation
μ = 0.09 mm^−1^
*T* = 291 (2) K
0.31 × 0.31 × 0.19 mm

#### Data collection

Rigaku R-AXIS RAPID diffractometer
Absorption correction: multi-scan (*ABSCOR*; Higashi, 1995 ▶) *T*_min_ = 0.971, *T*_max_ = 0.982
8527 measured reflections
3911 independent reflections
2370 reflections with *I* > 2σ(*I*)
*R*_int_ = 0.028

#### Refinement

*R*[*F* ^2^ > 2σ(*F* ^2^)] = 0.057
*wR*(*F* ^2^) = 0.195
*S* = 1.05
3911 reflections
220 parameters
H-atom parameters constrained
Δρ_max_ = 0.20 e Å^−3^
Δρ_min_ = −0.27 e Å^−3^

### 

Data collection: *RAPID-AUTO* (Rigaku, 1998 ▶); cell refinement: *RAPID-AUTO*; data reduction: *CrystalStructure* (Rigaku/MSC, 2002 ▶); program(s) used to solve structure: *SHELXS97* (Sheldrick, 2008 ▶); program(s) used to refine structure: *SHELXL97* (Sheldrick, 2008 ▶); molecular graphics: *SHELXTL* (Sheldrick, 2008 ▶); software used to prepare material for publication: *SHELXL97*.

## Supplementary Material

Crystal structure: contains datablocks global, I. DOI: 10.1107/S160053680802240X/cv2422sup1.cif

Structure factors: contains datablocks I. DOI: 10.1107/S160053680802240X/cv2422Isup2.hkl

Additional supplementary materials:  crystallographic information; 3D view; checkCIF report

## Figures and Tables

**Table 1:** Hydrogen-bond geometry (Å, °)

*D*—H⋯*A*	*D*—H	H⋯*A*	*D*⋯*A*	*D*—H⋯*A*
O4—H22⋯O2	0.85	1.94	2.789 (3)	176
O4—H21⋯O1^i^	0.85	2.02	2.751 (3)	143
O3—H2⋯O4^ii^	0.82	1.84	2.658 (3)	173
O2—H6⋯N2^iii^	0.82	1.84	2.636 (3)	164
O1—H4⋯N4^iv^	0.82	1.79	2.596 (3)	170

Symmetry codes: (i) 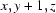; (ii) 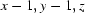; (iii) 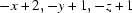; (iv) 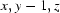.

**Fig. 1.**
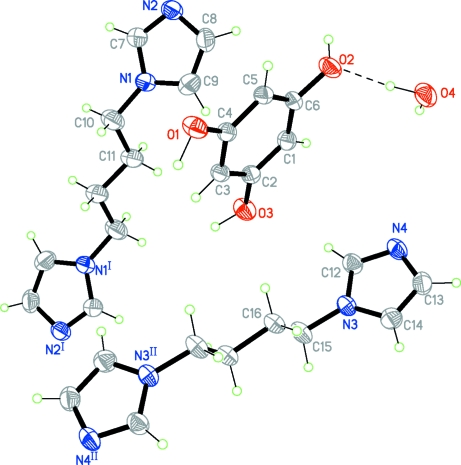
A portion of the crystal structure of (I), showing the atomic numbering and 30% probability displacement ellipsoids [symmetry code: (I) -x, -y, -z + 1; (II) -x, -y, -z]. Dashed line indicates the hydrogen-bonding interaction.

**Fig. 2.**
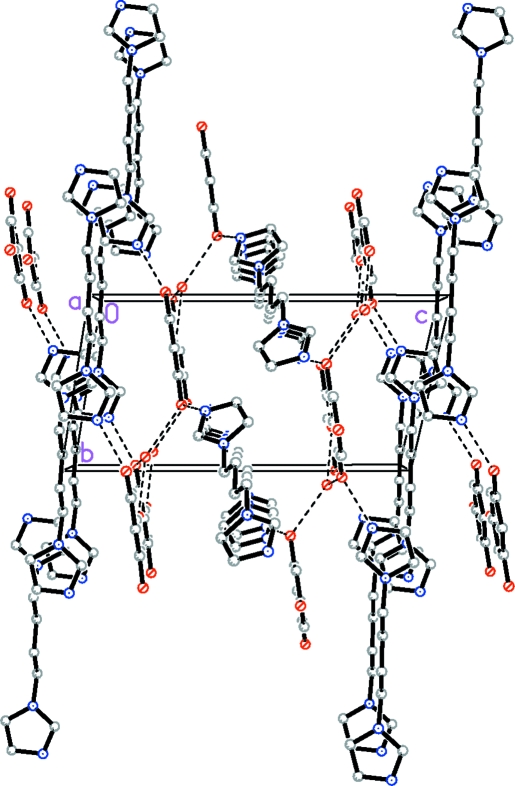
A partial packing view, showing the three-dimensional hydrogen-bonding network. Dashed lines indicate the hydrogen-bonding interactions. H atoms have been omitted for clarity.

## supplementary crystallographic information

### Comment

The 1,1'-(1,4-butanediyl)diimidazole can be used as a flexible ligand to construct coordination polymer materials (Ma *et al.*, 2003; Yu *et al.*, 2008). In this paper, we report the new title compound, (I), synthesized by the reaction of 1,1'-(1,4-butanediyl)diimidazole and *m*-trihydroxybenzene in an methanol solution.

The asymmetric unit of the title compound, C_10_H_14_N_4_.C_6_H_6_O_3_.H_2_O, contains one molecule of benzene-1,3,5-triol, two halfs of two independent centrosymmetric molecules of 1,1'-butane-1,4-diyldi-1*H*-imidazole and one crystalline water molecule (Figure 1). The two 1,1'-(1,4-butanediyl)diimidazole molecules both lie on inversion center.

There are five symmetry independent 'active' H atoms in the crystal structure; all of them participate in hydrogen bonds, which link the 1,1'-(1,4-butanediyl)diimidazole molecules, *m*-trihydroxybenzene molecule and water solvent molecule into an infinite three-dimensional network (Table 1, Figure 2).

### Experimental

1,1'-(1,4-Butanediyl)diimidazole was prepared from imidazole and 1,4-dibromobutane in dimethylsulfoxide solution (Ma *et al.*, 2003). *m*-trihydroxybenzene (0.126 g, 1 mmol) and 1,1'-(1,4-butanediyl)diimidazole (0.380 g, 2 mmol) were dissolved in hot methanol solution (15 ml) then a clear solution was obtained. The resulting solution was allowed to stand in a desiccator at room temperature for several days. Red crystals of (I) were obtained.

### Refinement

H atoms bound to C atoms were placed in calculated positions and treated as riding on their parent atoms, with C—H = 0.93 Å (aromatic); C—H = 0.97 Å (methylene) and with *U*_iso_(H) = 1.2*U*_eq_(C). The hydroxy H atoms were placed in calculated positions and treated as riding on their parent atoms, with O—H = 0.82 Å and with *U*_iso_(H) = 1.5*U*_eq_(O). Water H atoms were initially located in a difference Fourier map, but they were treated as riding on their parent atoms with O—H = 0.85 Å and with *U*_iso_(H) = 1.5*U*_eq_(O).

### Crystal data

**Table:**

C_10_H_14_N_4_·C_6_H_6_O_3_·H_2_O	*Z* = 2
*M**_r_* = 334.38	*F*_000_ = 356
Triclinic, *P*1	*D*_x_ = 1.289 Mg m^−^^3^
Hall symbol: -P 1	Mo *K*α radiation λ = 0.71073 Å
*a* = 7.964 (5) Å	Cell parameters from 5362 reflections
*b* = 8.405 (7) Å	θ = 3.1–27.6º
*c* = 14.800 (9) Å	µ = 0.09 mm^−^^1^
α = 98.40 (3)º	*T* = 291 (2) K
β = 92.93 (2)º	Block, red
γ = 117.47 (3)º	0.31 × 0.31 × 0.19 mm
*V* = 861.5 (10) Å^3^	

### Data collection

**Table:**

Rigaku R-AXIS RAPID diffractometer	3911 independent reflections
Radiation source: fine-focus sealed tube	2370 reflections with *I* > 2σ(*I*)
Monochromator: graphite	*R*_int_ = 0.028
*T* = 291(2) K	θ_max_ = 27.5º
ω scans	θ_min_ = 3.1º
Absorption correction: multi-scan(ABSCOR; Higashi, 1995)	*h* = −10→10
*T*_min_ = 0.971, *T*_max_ = 0.982	*k* = −10→10
8527 measured reflections	*l* = −18→19

### Refinement

**Table:**

Refinement on *F*^2^	Secondary atom site location: difference Fourier map
Least-squares matrix: full	Hydrogen site location: inferred from neighbouring sites
*R*[*F*^2^ > 2σ(*F*^2^)] = 0.057	H-atom parameters constrained
*wR*(*F*^2^) = 0.195	*w* = 1/[σ^2^(*F*_o_^2^) + (0.1078*P*)^2^ + 0.0441*P*] where *P* = (*F*_o_^2^ + 2*F*_c_^2^)/3
*S* = 1.05	(Δ/σ)_max_ < 0.001
3911 reflections	Δρ_max_ = 0.20 e Å^−^^3^
220 parameters	Δρ_min_ = −0.27 e Å^−^^3^
Primary atom site location: structure-invariant direct methods	Extinction correction: none

### Special details

**Table:**

Geometry. All e.s.d.'s (except the e.s.d. in the dihedral angle between two l.s. planes) are estimated using the full covariance matrix. The cell e.s.d.'s are taken into account individually in the estimation of e.s.d.'s in distances, angles and torsion angles; correlations between e.s.d.'s in cell parameters are only used when they are defined by crystal symmetry. An approximate (isotropic) treatment of cell e.s.d.'s is used for estimating e.s.d.'s involving l.s. planes.
Refinement. Refinement of *F*^2^ against ALL reflections. The weighted *R*-factor *wR* and goodness of fit *S* are based on *F*^2^, conventional *R*-factors *R* are based on *F*, with *F* set to zero for negative *F*^2^. The threshold expression of *F*^2^ > σ(*F*^2^) is used only for calculating *R*-factors(gt) *etc*. and is not relevant to the choice of reflections for refinement. *R*-factors based on *F*^2^ are statistically about twice as large as those based on *F*, and *R*- factors based on ALL data will be even larger.

### Fractional atomic coordinates and isotropic or equivalent isotropic displacement parameters (Å^2^)

**Table:**

	*x*	*y*	*z*	*U*_iso_*/*U*_eq_	
C1	0.6082 (3)	0.4322 (3)	0.27525 (15)	0.0495 (5)	
H1	0.5935	0.5359	0.2907	0.059*	
C2	0.4512 (3)	0.2634 (3)	0.24735 (14)	0.0463 (5)	
C3	0.4707 (3)	0.1074 (3)	0.22121 (14)	0.0440 (5)	
H3	0.3639	−0.0060	0.2009	0.053*	
C4	0.6534 (3)	0.1248 (3)	0.22616 (14)	0.0431 (5)	
C5	0.8124 (3)	0.2923 (3)	0.25642 (14)	0.0445 (5)	
H5	0.9340	0.3024	0.2609	0.053*	
C6	0.7880 (3)	0.4458 (3)	0.28012 (14)	0.0461 (5)	
C7	0.5669 (4)	0.2164 (4)	0.62310 (17)	0.0599 (6)	
H7	0.5388	0.1550	0.6722	0.072*	
C8	0.7113 (4)	0.4008 (4)	0.5372 (2)	0.0807 (9)	
H8	0.8061	0.4952	0.5144	0.097*	
C9	0.5333 (4)	0.2938 (5)	0.4948 (2)	0.0814 (9)	
H9	0.4827	0.2997	0.4381	0.098*	
C10	0.2441 (4)	0.0249 (4)	0.5326 (2)	0.0770 (8)	
H10	0.2144	−0.0270	0.5878	0.092*	
H11	0.2360	−0.0698	0.4837	0.092*	
C11	0.0998 (3)	0.0796 (4)	0.50659 (19)	0.0655 (7)	
H12	0.1248	0.1263	0.4498	0.079*	
H13	0.1094	0.1768	0.5543	0.079*	
C12	0.2720 (3)	0.5418 (3)	0.10952 (16)	0.0561 (6)	
H16	0.2520	0.5172	0.1684	0.067*	
C13	0.3851 (4)	0.6740 (4)	−0.00145 (17)	0.0606 (6)	
H14	0.4595	0.7618	−0.0344	0.073*	
C14	0.2445 (4)	0.5074 (4)	−0.03878 (17)	0.0613 (6)	
H15	0.2052	0.4582	−0.1013	0.074*	
C15	0.0159 (3)	0.2379 (3)	0.02391 (18)	0.0572 (6)	
H17	−0.0806	0.2134	−0.0265	0.069*	
H18	−0.0429	0.2277	0.0801	0.069*	
C16	0.0830 (3)	0.0959 (3)	0.00706 (15)	0.0502 (6)	
H19	0.1731	0.1147	0.0593	0.060*	
H20	0.1485	0.1102	−0.0471	0.060*	
N1	0.4412 (3)	0.1758 (3)	0.54990 (13)	0.0574 (5)	
N2	0.7338 (3)	0.3514 (3)	0.61894 (15)	0.0661 (6)	
N3	0.1703 (3)	0.4238 (2)	0.03210 (12)	0.0497 (5)	
N4	0.4031 (3)	0.6959 (3)	0.09239 (14)	0.0573 (5)	
O1	0.6793 (2)	−0.0244 (2)	0.20167 (12)	0.0587 (5)	
H4	0.5847	−0.1054	0.1677	0.088*	
O2	0.9405 (2)	0.6159 (2)	0.30773 (14)	0.0675 (5)	
H6	1.0348	0.6068	0.3247	0.101*	
O3	0.2755 (2)	0.2535 (2)	0.24808 (13)	0.0643 (5)	
H2	0.1939	0.1464	0.2426	0.096*	
O4	0.9883 (2)	0.9172 (3)	0.23236 (15)	0.0838 (6)	
H21	0.8952	0.9328	0.2485	0.101*	
H22	0.9716	0.8225	0.2530	0.101*	

### Atomic displacement parameters (Å^2^)

**Table:**

	*U*^11^	*U*^22^	*U*^33^	*U*^12^	*U*^13^	*U*^23^
C1	0.0515 (13)	0.0396 (12)	0.0590 (13)	0.0239 (11)	0.0033 (10)	0.0078 (10)
C2	0.0425 (11)	0.0467 (13)	0.0518 (12)	0.0225 (10)	0.0043 (9)	0.0113 (9)
C3	0.0374 (10)	0.0401 (12)	0.0503 (11)	0.0165 (9)	−0.0001 (9)	0.0055 (9)
C4	0.0432 (11)	0.0403 (12)	0.0464 (11)	0.0210 (10)	0.0037 (9)	0.0067 (9)
C5	0.0380 (11)	0.0443 (13)	0.0502 (11)	0.0192 (10)	0.0014 (9)	0.0090 (9)
C6	0.0416 (11)	0.0352 (12)	0.0532 (12)	0.0118 (10)	0.0007 (9)	0.0086 (9)
C7	0.0559 (14)	0.0609 (16)	0.0589 (14)	0.0246 (13)	0.0035 (11)	0.0120 (12)
C8	0.0605 (17)	0.069 (2)	0.117 (2)	0.0253 (16)	0.0187 (17)	0.0479 (18)
C9	0.074 (2)	0.096 (2)	0.0739 (17)	0.0352 (19)	0.0028 (15)	0.0350 (16)
C10	0.0540 (16)	0.0608 (18)	0.104 (2)	0.0216 (14)	−0.0041 (15)	0.0053 (15)
C11	0.0538 (14)	0.0644 (17)	0.0701 (15)	0.0261 (13)	−0.0046 (12)	0.0007 (13)
C12	0.0588 (14)	0.0445 (14)	0.0537 (13)	0.0177 (12)	0.0049 (11)	0.0011 (10)
C13	0.0570 (14)	0.0543 (16)	0.0706 (16)	0.0231 (13)	0.0136 (12)	0.0216 (12)
C14	0.0626 (15)	0.0632 (17)	0.0537 (13)	0.0266 (14)	0.0070 (12)	0.0095 (12)
C15	0.0451 (12)	0.0400 (13)	0.0717 (15)	0.0117 (10)	0.0073 (11)	−0.0024 (11)
C16	0.0418 (12)	0.0415 (13)	0.0559 (12)	0.0134 (10)	0.0030 (10)	0.0000 (10)
N1	0.0483 (11)	0.0548 (13)	0.0616 (12)	0.0200 (10)	−0.0006 (9)	0.0080 (10)
N2	0.0545 (13)	0.0524 (13)	0.0832 (15)	0.0220 (11)	−0.0070 (11)	0.0064 (11)
N3	0.0477 (10)	0.0376 (11)	0.0563 (11)	0.0159 (9)	0.0070 (9)	0.0026 (8)
N4	0.0512 (11)	0.0358 (11)	0.0724 (13)	0.0131 (9)	0.0036 (10)	0.0020 (9)
O1	0.0444 (9)	0.0438 (9)	0.0818 (12)	0.0222 (8)	−0.0023 (8)	−0.0069 (8)
O2	0.0494 (10)	0.0371 (9)	0.1014 (13)	0.0126 (8)	−0.0109 (9)	0.0056 (9)
O3	0.0432 (9)	0.0548 (11)	0.0984 (13)	0.0274 (8)	0.0079 (9)	0.0107 (10)
O4	0.0461 (10)	0.0723 (14)	0.1375 (18)	0.0247 (10)	0.0120 (11)	0.0456 (12)

### Geometric parameters (Å, °)

**Table:**

C1—C2	1.373 (3)	C11—C11^i^	1.512 (5)
C1—C6	1.380 (3)	C11—H12	0.9700
C1—H1	0.9300	C11—H13	0.9700
C2—O3	1.364 (3)	C12—N4	1.306 (3)
C2—C3	1.392 (3)	C12—N3	1.336 (3)
C3—C4	1.390 (3)	C12—H16	0.9300
C3—H3	0.9300	C13—C14	1.335 (4)
C4—O1	1.364 (3)	C13—N4	1.365 (3)
C4—C5	1.378 (3)	C13—H14	0.9300
C5—C6	1.390 (3)	C14—N3	1.356 (3)
C5—H5	0.9300	C14—H15	0.9300
C6—O2	1.365 (3)	C15—N3	1.458 (3)
C7—N2	1.302 (3)	C15—C16	1.512 (3)
C7—N1	1.326 (3)	C15—H17	0.9700
C7—H7	0.9300	C15—H18	0.9700
C8—C9	1.334 (4)	C16—C16^ii^	1.516 (4)
C8—N2	1.364 (4)	C16—H19	0.9700
C8—H8	0.9300	C16—H20	0.9700
C9—N1	1.347 (4)	O1—H4	0.8200
C9—H9	0.9300	O2—H6	0.8200
C10—N1	1.471 (3)	O3—H2	0.8200
C10—C11	1.475 (4)	O4—H21	0.8501
C10—H10	0.9700	O4—H22	0.8500
C10—H11	0.9700		
			
C2—C1—C6	119.1 (2)	C10—C11—H13	109.4
C2—C1—H1	120.4	C11^i^—C11—H13	109.4
C6—C1—H1	120.4	H12—C11—H13	108.0
O3—C2—C1	117.6 (2)	N4—C12—N3	111.8 (2)
O3—C2—C3	121.2 (2)	N4—C12—H16	124.1
C1—C2—C3	121.2 (2)	N3—C12—H16	124.1
C4—C3—C2	118.5 (2)	C14—C13—N4	109.7 (2)
C4—C3—H3	120.7	C14—C13—H14	125.1
C2—C3—H3	120.7	N4—C13—H14	125.1
O1—C4—C5	118.36 (19)	C13—C14—N3	106.8 (2)
O1—C4—C3	120.58 (19)	C13—C14—H15	126.6
C5—C4—C3	121.07 (19)	N3—C14—H15	126.6
C4—C5—C6	118.88 (19)	N3—C15—C16	112.84 (19)
C4—C5—H5	120.6	N3—C15—H17	109.0
C6—C5—H5	120.6	C16—C15—H17	109.0
O2—C6—C1	117.4 (2)	N3—C15—H18	109.0
O2—C6—C5	121.5 (2)	C16—C15—H18	109.0
C1—C6—C5	121.1 (2)	H17—C15—H18	107.8
N2—C7—N1	112.9 (2)	C15—C16—C16^ii^	111.3 (2)
N2—C7—H7	123.6	C15—C16—H19	109.4
N1—C7—H7	123.6	C16^ii^—C16—H19	109.4
C9—C8—N2	110.0 (3)	C15—C16—H20	109.4
C9—C8—H8	125.0	C16^ii^—C16—H20	109.4
N2—C8—H8	125.0	H19—C16—H20	108.0
C8—C9—N1	106.8 (3)	C7—N1—C9	106.2 (2)
C8—C9—H9	126.6	C7—N1—C10	125.6 (2)
N1—C9—H9	126.6	C9—N1—C10	128.2 (2)
N1—C10—C11	113.9 (2)	C7—N2—C8	104.1 (2)
N1—C10—H10	108.8	C12—N3—C14	106.6 (2)
C11—C10—H10	108.8	C12—N3—C15	127.4 (2)
N1—C10—H11	108.8	C14—N3—C15	126.0 (2)
C11—C10—H11	108.8	C12—N4—C13	105.2 (2)
H10—C10—H11	107.7	C4—O1—H4	109.5
C10—C11—C11^i^	111.3 (3)	C6—O2—H6	109.5
C10—C11—H12	109.4	C2—O3—H2	109.5
C11^i^—C11—H12	109.4	H21—O4—H22	102.9

Symmetry codes: (i) −*x*, −*y*, −*z*+1; (ii) −*x*, −*y*, −*z*.

### Hydrogen-bond geometry (Å, °)

**Table:**

*D*—H···*A*	*D*—H	H···*A*	*D*···*A*	*D*—H···*A*
O4—H22···O2	0.85	1.94	2.789 (3)	176
O4—H21···O1^iii^	0.85	2.02	2.751 (3)	143
O3—H2···O4^iv^	0.82	1.84	2.658 (3)	173
O2—H6···N2^v^	0.82	1.84	2.636 (3)	164
O1—H4···N4^vi^	0.82	1.79	2.596 (3)	170

Symmetry codes: (iii) *x*, *y*+1, *z*; (iv) *x*−1, *y*−1, *z*; (v) −*x*+2, −*y*+1, −*z*+1; (vi) *x*, *y*−1, *z*.

## References

[bb1] Higashi, T. (1995). *ABSCOR* Rigaku Corporation, Tokyo, Japan.

[bb2] Ma, J.-F., Yang, J., Zheng, G.-L. & Liu, J.-F. (2003). *Inorg. Chem.* **42**, 7531–7534. 10.1021/ic034846q 14606848

[bb3] Rigaku (1998). *RAPID-AUTO* Rigaku Corporation, Tokyo, Japan.

[bb4] Rigaku/MSC (2002). *CrystalStructure* Rigaku/MSC Inc., The Woodlands, Texas, USA.

[bb5] Sheldrick, G. M. (2008). *Acta Cryst.* A**64**, 112–122. 10.1107/S0108767307043930 18156677

[bb6] Yu, Y.-H., Shi, A.-E., Su, Y., Hou, G.-F. & Gao, J.-S. (2008). *Acta Cryst.* E**64**, m628. 10.1107/S160053680800874X PMC2961250 21202182

